# Dengue Fever among Patients Visiting the Outpatient Department of Dermatology in a Tertiary Care Centre: A Descriptive Cross-sectional Study

**DOI:** 10.31729/jnma.8059

**Published:** 2023-06-30

**Authors:** Eliz Aryal, Raunak Bista, Prashanna Raj Shrestha, Garima Regmi

**Affiliations:** 1 Department of Dermatology, Kathmandu Medical College and Teaching Hospital, Sinamangal, Kathmandu, Nepal

**Keywords:** *dengue fever*, *prevalence*, *rash*

## Abstract

**Introduction:**

Dengue is in an increasing trend in our part of the world mostly due to global warming, It can present with various manifestations including cutaneous manifestations. The main objective of our study was to find out the prevalence of dengue fever among patients visiting the Outpatient Department of Dermatology in a tertiary care centre.

**Methods:**

A descriptive cross-sectional study was conducted among patients visiting the Outpatient Department of Dermatology in a tertiary care centre after taking ethical approval from the Institutional Review Committee (Reference number: 09092022\04). Data from 1 June 2022 to 8 September 2022 were collected between 1 December 2022 to 20 February 2023 from the hospital records. The laboratory data of individuals were analyzed to find out the prevalence of dengue fever. A Visual Analogue Scale was used to assess the severity of pruritus. Convenience sampling method was used. Point estimate and 95% Confidence Interval were calculated.

**Results:**

Among 7442 patients, dengue fever was found to be in 202 (2.71%) (2.34-3.08, 95% Confidence Interval) patients. The mean duration of fever was 3.02±2.960 days. The mean duration of onset of rash after having a fever was 2.56±2.032 days. The most common cutaneous manifestation was maculopapular rash 70 (34.65%).

**Conclusions:**

The prevalence of dengue fever was found to be lower than in the studies done in similar settings.

## INTRODUCTION

Dengue fever (DF), caused by the dengue virus (DENV) is a single-stranded RNA flavivirus of the family Flaviviridae.^1^ Mild form of DF includes fever, headache, retro-orbital pain, myalgia, and arthralgia with varied skin rash and severe conditions including dengue hemorrhagic fever and dengue shock syndrome.^2^

The first reported incidence of dengue was reported in Nepal in 2004 and was considered to be imported from India, based on genetic similarity. Population growth, rapid urbanisation, increase in international travel from endemic areas and global warming are playing a major role in disease spread.^3^ Cutaneous manifestations of dengue are present in 65% of patients. Skin lesions may be the first symptom of DF, and can be helpful for making the diagnosis.^3^

The aim of the study was to find out the prevalence of dengue fever among patients visiting the Outpatient Department of Dermatology in a tertiary care centre.

## METHODS

This descriptive cross-sectional study was conducted in the Department of Dermatology, Venereology and Leprology at Kathmandu Medical College Teaching Hospital after taking ethical approval from the Institutional Review Committee (Reference number: 09092022\04). Data from 1 June 2022 to 8 September 2022 were collected between 1 December 2022 to 20 February 2023 from the hospital records. All the suspected patients above 18 years who presented to the Department of Dermatology with complete hospital record data were included in the study after giving consent. Pregnant female and immunocompromised patients were excluded. Convenience sampling method was used. The sample size was calculated by using the following formula:


$$\begin{array}{ll} \text{n} & ={\text{Z}}^{2}\times \frac{\text{p}\times \text{q}}{{\text{e}}^{\text{2}}}\\ & =1.96^{2}\times \frac{0.484\times 0.516}{0.02^{2}}\\ & =2032 \end{array}$$

Where,

n = minimum required sample size
Z = 1.96 at 95% of confidence Interval (CI)
p = prevalence taken from a previous study, 48.40%^4^
q = 1-p
e = margin of error, 2%

The minimum required sample size was 2032. However, the final sample size taken was 7442.

The diagnosis of dengue was done with a chromatographic immunoassay kit for rapid and differential detection kit of immunoglobulin G (IgG) and immunoglobulin M (IgM) along with dengue specific antigen (NS1) against all types of dengue viruses using human blood.^5,6^ Demographic details of the patients including age, gender, education, site and duration of the lesion, and onset of fever were recorded. Mucocutaneous manifestation regarding, the type of rash, the onset of rash with fever and routine blood tests including complete blood count, and liver and renal function tests were recorded. Clinical manifestations including headache, retro-orbital pain, pruritus, burning sensation, and body aches were recorded. A visual analogue scale was used to measure the severity of pruritus.^5^

## RESULTS

Among 7442 patients, dengue fever was found to be in 202 (2.71%) (2.34-3.08, 95% CI) patients. There were 110 (54.45%) males and 92 (45.55%) females. The mean duration of fever was 3.02±2.960 days. The mean duration of onset of rash after having fever was 2.56±2.032 days.

The most common cutaneous manifestation was maculopapular rash 70 (34.65%) followed by morbilliform rash 69 (34.15%), petechiae rash 33 (16.34%) and urticarial rash 43 (21.29%). Around 35 (17.32%) patients had mixed cutaneous manifestations. Desquamation of skin was present in 25 (12.37%). The generalised burning sensation was present among 65 (32.18%). The burning sensation was felt more over hands and feet 20 (9.90%). The mucosa of the oral lesion was involved in 35 (17.33%) of the dengue patients (Table 1).

**Table 1:** Frequency of mucosal involvement (n= 202).

Mucosa involved	n (%)
Oral lesion	35 (17.33)
Nasal	10 (4.95)
Occular	8 (3.97)
Genital	5(2.48)

Eighty-seven (43.07%) patients had no history of fever before the onset of skin rash. The most predominant site for a skin rash was on the chest area 93 (46.04%) followed by the hand 88 (43.56%) (Table 2).

**Table 2:** Site of skin rash (n= 202).

Site	n (%)
Chest	93 (46.04)
Hand	88 (43.56)
Abdomen	86 (42.57)
Palm	75 (37.13)
Leg	59 (29.21)
Neck	57 (28.22)
Back	66 (32.67)
Sole	39 (19.31)
Face	24 (11.88)

**Figure 1:**
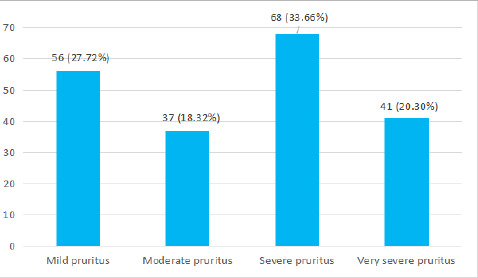
The severity of pruritis from VAS (n= 197).

Around 197 (97.52%) had experienced pruritus. Most of the patients had severe pruritus 68 (33.66%) (Figure 1).

## DISCUSSION

The prevalence of dengue was found to be 202 (2.71%) among 7442 patients. Rapid growth of population, urbinazation, global warning and increase used of nonbiodegradable products that severe as site of mosquito larva proliferation are the main cause for increasing in dengue fever.^6^ Ealier studies have proven monsoon and post monsoon months as a maximum activity of Aedes aegypti. This is due to presence of stagnating source of water following heavy rain fall, favoring breeding of mosquito vector.^7^ There is predominant male involvement of in our study. Studies conducted in other studies in Asia have reported a greater male incidence.^8,9^

Regarding the history of fever, 115 (56.9%) cases had fever before onset of rash. Around 50% of infected patients report of having rash during febrile period in a similar study. This is due to interaction of virus with host cell causing the release of chemical mediator and initiation of immunological mechanism.^10^

In many studies, generalized burning sensation mostly over hand and feet has being documented in various study and even we experienced similar results in our patients. This can be due to psychosocial factors, lack of proper nutrition at time of disease as well as different viral serotype. The most common mucosal involvement in our study was oral cavity with clinical feature of congestion of mucous membrane and bleeding gum. Similar pattern was observed in study done in similar settings.^11^

Cutaneous manifestation of dengue fever has been reported to occur in 80% of patients.^11,12^ A study done out of 124 cases, skin manifestation were seen in 46.8%.^12^ Maculopapular rash 70 (34.7%) was predominant in our study followed by morbilliform rash. Similar presentation was seen in Pakistan where maculopapular rash was seen in 31.7% and morbilliform rash was seen in 64.5%. In another study, similar rash was seen in 42.9% of total patients.^11^ A study mentions that macular popular rash was found in 1-6 days after onset of fever.^12^ Macular erythema was the earliest and most commonest observed in many study as due to capillary dilation and thought to be immune response to the virus.^11^ Whereas in another study, morbilliform rash was predominantly present.^13^ This difference could be due to variation in sampling method, population, racial and environment factors. The skin rash represents a reaction of vessels to cytokines with expression of inflammatory cell infiltration and dermal edema after increased permeability of vessel, that is produced by an intact immune system.^10^

Pruritus was most common clinical feature present in 197 (97.52%) in our study and was evaluated by using visual analog scales. In a similar study, generalized pruritus was observed in 69.2%.^14^

There were several limitations of our study. Since this is a single-centered study, the findings cannot be generalized. The study was only conducted in the outpatient department as a result severe cases, and complicated cases of dengue fever like hemorrhagic shock were not taken into account.

## CONCLUSIONS

The prevalence of dengue fever was found to be lower than in the studies done in similar settings. A variety of cutaneous features were observed in patients with dengue. Early recognition of mucocutaneous features is important, as it may progress to severe life-threatening conditions. As cases of dengue are increasing annually, further new recharge need to be done in the field of dermatology in Nepal.
